# Systematic review on the implementation of metrological assurance systems for medical devices in Latin America

**DOI:** 10.3389/fmed.2024.1281199

**Published:** 2024-06-21

**Authors:** Harold M. Farfán-Vargas, Dante Espinoza-Morriberon, Marcia M. Moya-Salazar, Hans Contreras-Pulache, Jeel Moya-Salazar

**Affiliations:** ^1^School of Biomedical Engineering, Faculty of Engineering, Universidad Tecnológica del Perú, Lima, Peru; ^2^Digital Transformation Center, Universidad Norbert Wiener, Lima, Peru; ^3^Education Unit, Nesh Hubbs, Lima, Peru

**Keywords:** metrological characterization, medical devices, Latin America, ISO 9001, quality assurance, ISO 17025, external quality assurance, reference methods

## Abstract

**Background:**

Metrology plays a crucial role in small healthcare service businesses to ensure the quality of products and services. While legal metrology in healthcare exists in some regions, it lacks harmonization. In other countries, there is limited presence of metrology in medical and biomedical engineering. We aimed to evaluate the implementation of metrological assurance systems for medical devices in Latin America.

**Methods:**

A systematic review was conducted following PRISMA 2020 guidelines and registered with PROSPERO (CRD42022359284). Searches were performed across 13 databases from October 30th to November 3rd, 2022. The search equation was “(((quality assurance) AND (metrology)) AND (medical devices)).” A total of 7,789 documents were identified, of which only 16 met the inclusion criteria.

**Results:**

The majority of studies (75%) were conducted in Colombia, with a significant portion being undergraduate theses. The primary normative references used in the analyzed studies were ISO 10012 and ISO 17025, with the majority (68.75%) relying on national legislation for their approach. One study in Colombia referenced eight standards, and one in Brazil analyzed user involvement in medical device management. Among the included studies, 56.25% were conducted in healthcare institutions, mainly clinics. Most studies provided implementation guidelines, with ISO 10012 being prominent, alongside ISO 17025, which implicitly addresses ISO 9001 elements. Global bias was low across all studies.

**Conclusion:**

Our results underscore the importance of metrological assurance in managing medical devices in Latin America. The utilization of international standards and national legislation illustrates the diverse approaches adopted by different institutions. Future research should focus on optimizing metrological practices to enhance quality and safety in healthcare.

## 1 Introduction

The rise of small enterprises is a global trend, and they encounter challenges in service delivery and product quality (1), and the healthcare service sector is not the exception. In this context, metrology plays a pivotal role, as modern professional development relies on accurate measurements to ensure high-quality products and services (2). Metrology is the science responsible for making measurements, assuring and maintaining units of measurement, as well as verifying periodic calibration of equipment (3). Within this science, there are divisions like legal metrology, which has legal implications, industrial metrology, used in manufacturing and production, and scientific metrology, focused on research and development of measurements (4).

Legal Metrology in Healthcare is a challenge present in several countries, including those within the European Union. Bošnjaković and Džemić (5), from the Institute of Metrology of Bosnia and Herzegovina, highlight that this area is not harmonized among different Union members, except for equipment covered by directives NAWI 2014/31/EU and MID 2014/32/EU (6). Thus, the implementation of a Legal Metrology system is left to the discretion of each member country. This situation was also observed in Portugal in 2011 (7). Nevertheless, it is noted that the European Union only allows the commercialization of medical equipment with the CE marking, guaranteeing compliance with metrological and quality standards upon factory exit, in compliance with prevailing regulations, especially 2007/47/EC. This measure was relevant in 2011 as the medical equipment industry employed nearly half a million people. However, Do Ceu Ferreira also states that, in her specific case, the requirements for quality metrological traceability are often disregarded.

However, the results presented by Ferreira and Matos (8) for the case of Portugal shed light on the concerning disparity between budgetary matters and metrology in public hospitals. Among the evaluated hospitals (35 responses received), only 34% performed some form of calibration, either internally or through an outsourced service. Additionally, hospitals and private clinics showed a 1.5 times higher prevalence in pursuing quality, opting for in-house calibration services. A concerning fact is that, in hospitals with over 1,100 installed devices, only around 40 units were calibrated in the last year. Furthermore, the question of dubious metrological traceability in calibrations conducted by external entities arises due to inadequate oversight. Despite Do Céu Ferreira’s assertion (7) about the strengths of the European Union in manufacturing and ensuring the metrological chain from the factory, it has been demonstrated that this continuity does not persist post-sale and deployment, as evidenced by Ferreira and Matos (8) and Bošnjaković and Džemić (5).

In the Latin American context, Andres et al. (9) address metrology applied in the medical field in Colombia, where a regulatory framework has been implemented to ensure quality in healthcare service provision. Colombian legislation stipulates requirements for registration, marketing, and health surveillance of medical equipment (10). It is precisely this approach, in the Colombian context, that infuses calibrations and metrology oversight with a sense of obligation. However, the study also illuminates the absence of standardized criteria and guidelines for conducting this monitoring effectively.

In Cuba, a different approach to metrological management is adopted, with large-scale integration supervised by the government, as indicated in the study by Albert and Téllez (11). The Ministry of Public Health in Cuba has implemented metrology by establishing the Calibration Network in 2019 and calibration laboratories in electromedicine. The plan incorporates international standards like ISO 9001 for document management and metrological control, aiming for future accreditations and a patient-centered approach. Additionally, the acquisition of national metrology standards is suggested if necessary. On the other hand, thanks to appropriate political and legislative support, Mejías et al. (12) presented their report on the metrology situation to the National Health System and the National Bureau of Standards. The report emphasized the importance of strengthening metrology as support for the National Service of Metrology and the network of electromedicine services, which already dealt with the maintenance, repair, and calibration of medical equipment but faced limitations due to a lack of professionals and resources (13).

In Peru, metrology is still lagging behind, and in fields such as medicine and biomedical engineering, it is nearly nonexistent (14). Despite the development of appropriate infrastructure and the presence of the National Institute of Quality (INACAL) as the regulatory and oversight body for metrology in Peru (15), there is an urgent need for businesses to achieve desired competitiveness by demonstrating high-quality standards (16). A set of factors, including the absence of regulations enforcing metrological control, the lack of companies providing biomedical calibration services, and the limited number of INACAL-accredited tests. For small healthcare service companies, metrology offers the potential to enhance measurement accuracy, thus ensuring the quality of the services and products they offer. However, it is crucial to understand quality assurance processes in metrology on a country-by-country basis in order to determine whether metrological practices are adequate to meet quality standards and fulfill patients’ needs.

The objective of this study was to evaluate the implementation of metrological assurance systems for medical devices in Latin America.

## 2 Materials and methods

### 2.1 Study design, data sources, and search strategy

This systematic review followed the Preferred Reporting Items for Systematic Reviews and Meta-Analyses (PRISMA) 2020 guidelines (17) and was registered with the International Prospective Register of Systematic Reviews (PROSPERO) under code: CRD42022359284. Searches were conducted across 13 databases (PubMed, Scopus, Web of Science, ScienceDirect, JSTOR, EMBASE, Scielo, Latindex, LILIACS, and EBSCO), Google Scholar, Dimensions, and ALICIA CONCyTec (Peruvian Thesis Repository) from October 30th to November 3rd, 2022. The search equation used was “(((quality assurance) AND (metrology)) AND (medical devices))” without limitations on time or language (Supplementary Data Sheet 1).

### 2.2 Inclusion and exclusion criteria

**Figure 1:**
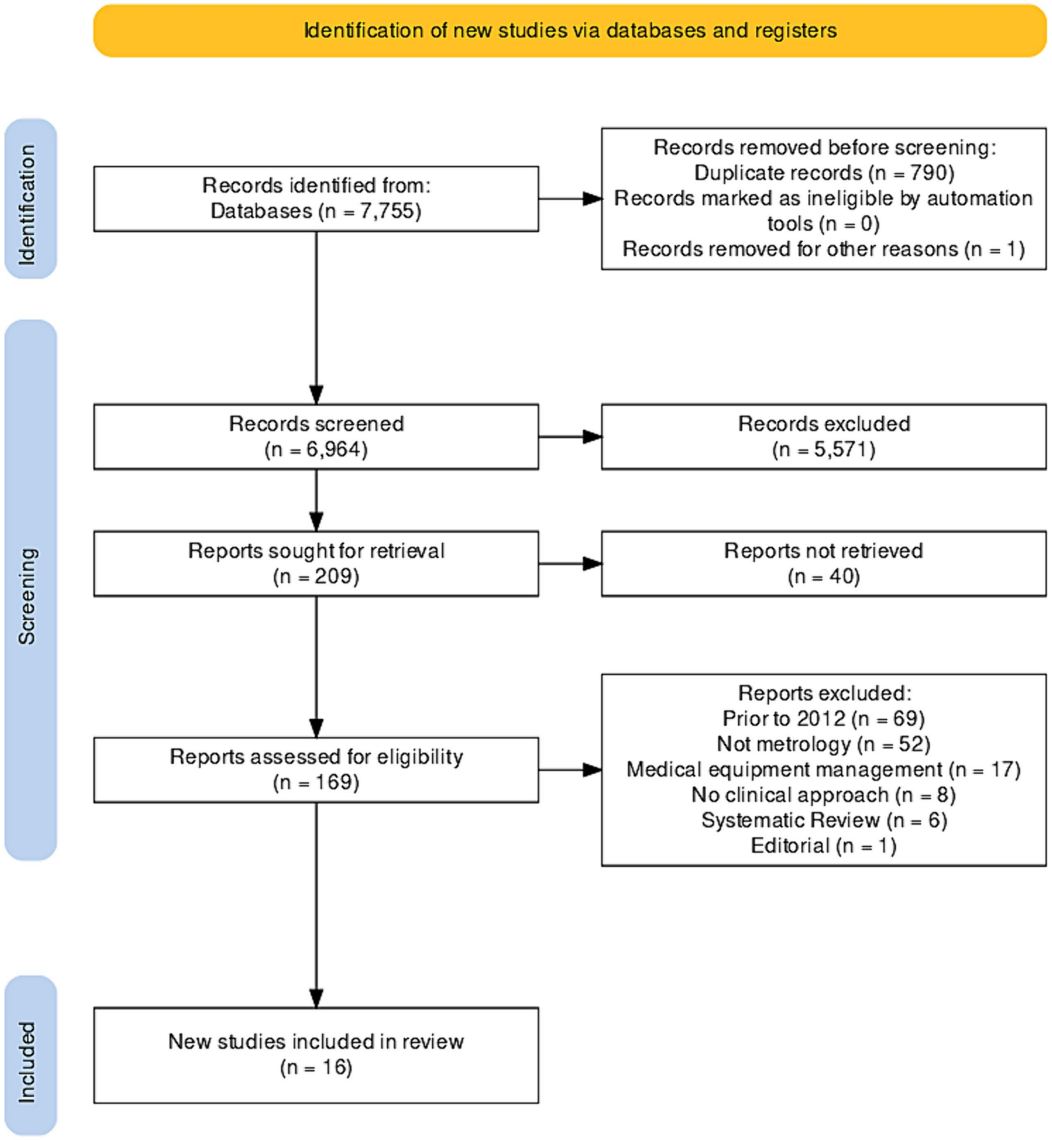
PRISMA flowchart of the selected studies.

Included studies met the following criteria: (i) original studies, clinical trials, validation studies, exploratory studies, commentaries, proceedings, and theses; (ii) publications between 2012 and 2022; (iii) articles in English, Portuguese, and Spanish; (iv) articles about medical devices; (v) studies in Latin American countries. Studies outside the clinical scope, systematic reviews, reflection articles, opinion letters, editorials, meta-analyses, documents focusing solely on calibration or medical equipment management were excluded (Figure 1).

### 2.3 Document selection and extraction

Two authors (HMF-V and JM-S) independently evaluated abstracts and excluded those not meeting inclusion criteria. Then, full-text reviews were performed according to a defined protocol for final analysis. Disagreements were resolved through consensus, following a previously reported procedure (18). Cohen’s Kappa correlation analysis was conducted to determine overall agreement between reviewers.

### 2.4 Data extraction, quality assessment, and data analysis

Data were extracted using the CASPe (Critical Appraisal Skills Programme) data matrix template in MS-Excel 2013 (Microsoft Corp., Redmond, WA, United States) to collect necessary information from systematic reviews. Cochrane’s risk of bias tool (Robvis 2.0) was used to assess bias, with studies not contributing to the study objective (analyzing at least one variable) considered to have a high risk of confusion (Figure 1). Disagreements among authors were resolved through consensus. Descriptive analysis of included studies was conducted using IBM SPSS version 23.0 (Armonk, NY, United States), estimating frequencies for categorical data and measures of central tendency for continuous data.

## 3 Results

**Figure 2:**
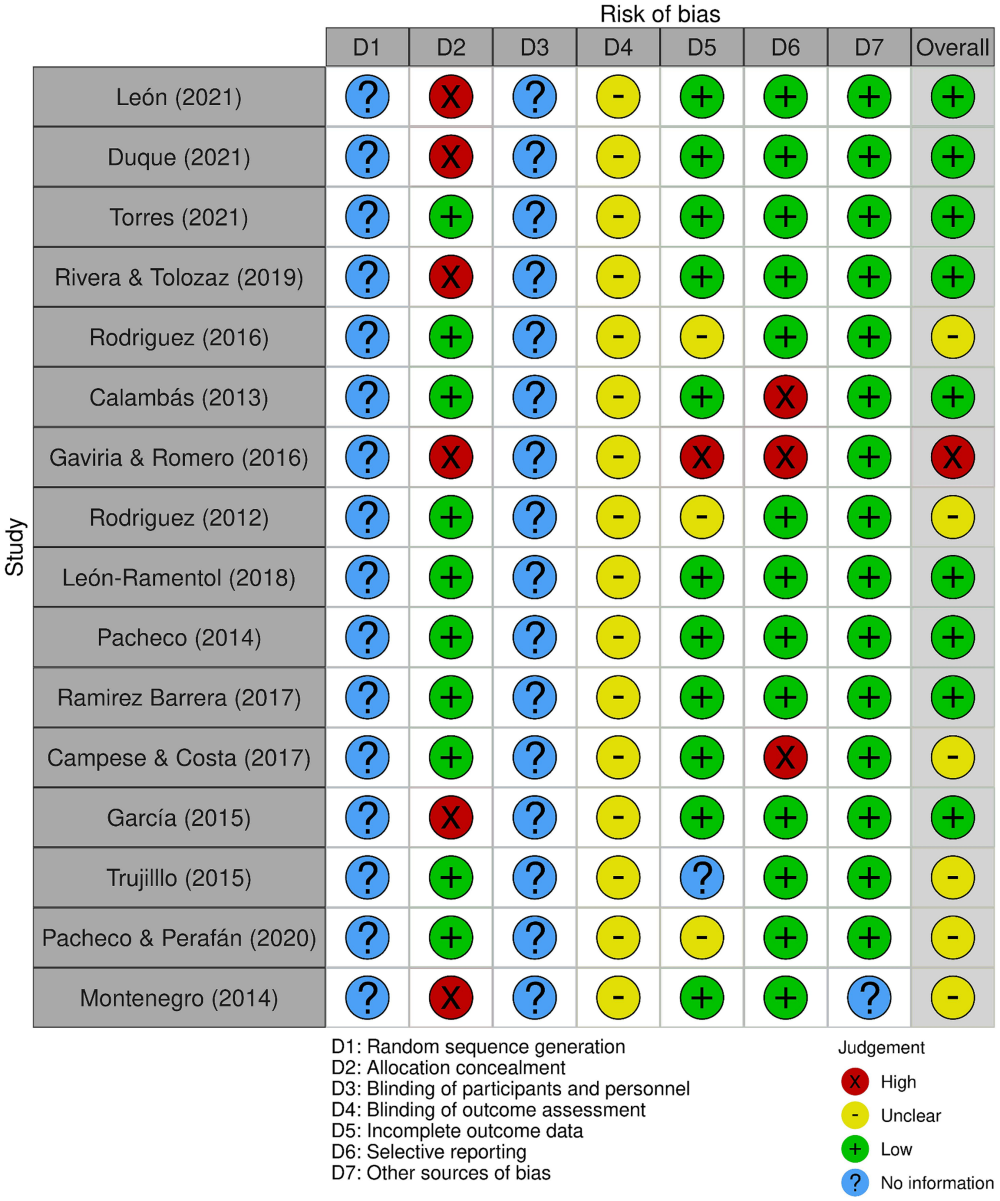
Bias analysis of the selected studies.

Throughout the research process, a total of 7,789 documents were found in Scopus (*n* = 9), PubMed (*n* = 23), JSTOR (*n* = 795), ScienceDirect (*n* = 489), Dimensions (*n* = 541), Alicia CONCyTec (*n* = 5), and Google Scholar (*n* = 5,893). No studies were identified in Scielo and LILACS, and 790 duplicate files were removed. Additionally, 5,571 documents not closely related to the topic were eliminated, leaving 209 documents for further analysis. However, it was not possible to retrieve 40 of these documents, resulting in the inclusion of 16 documents in this systematic review (19–34). Bias analysis is shown in Figure 2.

### 3.1 Document characteristics

Out of the 16 documents, 75% (12/16) were studies conducted in Colombia, with comparable numbers between Bogotá, Cali, and Medellín (19–21, 23–25, 27–30, 32, 33), and of these, 50% (6/12) were undergraduate theses (20, 21, 28–30, 33). Also, 18.7% (3/16) of the documents were published conference papers from Brazil (26), Colombia (23), and Cuba (29). A quarter of the documents were research articles (19, 24, 27, 31), and the majority (3/6) were published in 2021 (32–34) (Table 1).

**Table 1:** Bibliometric characteristics of the included studies.

Author	Year	Country	City	Type of study
Rodríguez (19)	2012	Colombia	Cali	Research article
Montenegro (20)	2013	Colombia	Cali	Undergraduate thesis
Calambás (21)	2014	Colombia	Cali	Undergraduate thesis
Pacheco (22)	2014	Costa Rica	San José	Research article
García et al. (23)	2015	Colombia	Medellín	Published conference
Trujillo et al. (24)	2015	Colombia	Medellín	Research article
Rodríguez et al. (25)	2016	Colombia	Cali	Book chapter
Campese and Costa (26)	2017	Brazil	Sao Paolo	Published conference
Ramírez Barrera et al. (27)	2017	Colombia	Medellín	Research article
Gaviria and Romero (28)	2018	Colombia	Bogotá	Tesis pregrado
León-Ramentol et al. (29)	2018	Cuba	Camagüey	Published conference
Rivera and Toloza (30)	2020	Colombia	Bogotá	Undergraduate thesis
Pacheco and Perafán (31)	2020	Ecuador	Guayaquil	Undergraduate thesis
Duque (32)	2021	Colombia	Bogotá	Postgraduate thesis
Torres (33)	2021	Colombia	Medellín	Undergraduate thesis
León (34)	2021	Colombia	Bogotá	Undergraduate thesis

### 3.2 Normative references used in different studies

Among the analyzed documents, various applicable standards for medical devices are mentioned (Table 2), such as IEC 60601–1-6 (standard to ensure basic safety and essential performance requirements for electromedical devices) and IEC 62366 (standard for applying usability engineering to medical devices) (26). Additionally, standards applicable to quality management like ISO 9000 (19, 25, 33), ISO 9001 (29–31), and ISO 15189 (specific to clinical laboratories) (29) are referenced, along with ISO 17000, which establishes vocabulary (33), ISO 17025 (specific to calibration and testing laboratories) (19, 21, 23, 25, 29–31, 33), and ISO 10012 (applied to measurement management systems) (19, 22, 23, 25, 27, 29, 31, 33).

**Table 2:** Standards cited in each included study.

Author	ANSI EQ56 2008 R 2013	ISO 5725	ISO 9000	ISO 9001	ISO 9241-11	ISO 10012	ISO 12000	ISO 13485	ISO 14971	ISO 15189	ISO 17000	ISO 17025	IEC 60601–1-6	IEC 62353	IEC 62366	Based on national legislation
León (34)	No	No	No	No	No	Yes	No	No	No	No	No	No	No	No	No	Yes
Duque (32)	No	No	No	No	No	No	No	No	Yes	No	No	No	No	No	No	Yes
Rivera and Toloza (30)	No	No	No	Yes	No	No	No	No	No	No	No	Yes	No	No	No	Yes
Gaviria and Romero (28)	No	No	No	No	No	No	No	No	No	No	No	No	No	No	No	No
Rodríguez et al. (25)	No	No	Yes	No	No	Yes	No	No	No	No	No	Yes	No	No	No	Yes
Calambás (21)	No	No	No	No	No	No	No	No	No	No	No	Yes	No	No	No	No
Montenegro (20)	No	No	No	No	No	No	No	Yes	Yes	No	No	No	No	No	No	No
Rodríguez (19)	No	No	Yes	No	No	Yes	No	No	No	No	No	Yes	No	No	No	Yes
León-Ramentol et al. (29)	No	No	No	Yes	No	No	No	No	No	Yes	No	Yes	No	No	No	Yes
Pacheco and Perafán (31)	No	No	No	Yes	No	Yes	No	No	No	No	No	Yes	No	No	No	Yes
Torres (33)	No	Yes	Yes	Yes	No	Yes	No	Yes	No	No	Yes	Yes	No	Yes	No	No
Ramírez Barrera et al. (27)	No	No	No	No	No	Yes	No	No	No	No	No	No	No	No	No	Yes
García et al. (23)	No	No	No	No	No	Yes	No	No	No	No	No	Yes	No	No	No	Yes
Trujillo et al. (24)	Yes	No	No	No	No	No	No	No	No	No	No	No	No	No	No	Yes
Pacheco (22)	No	No	No	No	No	Yes	Yes	No	No	No	No	No	No	No	No	Yes
Campese and Costa (26)	No	No	No	No	Yes	No	No	No	Yes	No	No	No	Yes	No	Yes	Yes

Furthermore, ANSI/AAMI EQ56 2008 R (2013), a standard providing recommendations for implementing a medical equipment management program, is mentioned in half of the documents (24). Half of the documents (8/16) mentions the use of ISO 10012 (19, 22, 23, 25, 27, 29, 31, 33) and the ISO 17025 standard (19, 21, 23, 25, 29–31, 33). The fourth part of the documents mentions the use of de ISO 9001 (29–31), 18.75% (3/16) identify the use of ISO 9000 (19, 25, 33) and ISO 14971 (20, 26, 32). Out of the 16 documents, 68.75% (11/16) uses the national legislation for their framework (20, 22, 23, 26–30, 32–34) and only a study (6.25%) mentions international legislation (24).

A significant work by Torres (33) stands out due to its reference to eight standards, including ISO 5725, ISO 9000, ISO 9001, ISO 10012, ISO 13485, ISO 17000, ISO 17025, and IEC 62353. This study focuses on optimizing the metrological assurance plan for the Departmental Public Health Laboratory of Antioquia, Colombia, and stands out for utilizing the highest number of international and national standards in its analysis. The work conducted by Campese and Costa (26) addresses user involvement in medical device management in small enterprises, particularly emphasizing safety factors for users. While briefly mentioning the effects of metrology in this context, its primary focus is on technical standards like IEC 60601–1-6 and IEC 62366, as well as the ergonomics standard ISO 9241-11.

The study by Gaviria and Romero (28) is notable for its use of national legislation instead of international standards, although paradoxically, it references images from ISO 17025 in its theoretical framework. The study entitled “Diseño e implementación de un plan de aseguramiento metrológico para equipos biomédicos de la Clínica Colsubsidio Calle 100 y el Centro Médico de Especialistas de la Calle 63” (Design and Implementation of a Metrological Assurance Plan for Biomedical Equipment at Clínica Colsubsidio Calle 100 and Centro Médico de Especialistas de la Calle 63) is interesting due to its focus on defining metrological needs for each listed piece of equipment and selecting patterns for calibration.

### 3.3 Analysis by type of organization

Table 3 describes the types of organizations in the included studies. Out of all the studies, 56.25% (9/16) were conducted in healthcare institutions (20, 21, 23, 28–30, 32–34), with the majority of these being carried out in clinics (20, 23, 28, 30, 32, 34), two in clinical laboratories (21, 33), and one in a university laboratory (29).

**Table 3:** Description of the work performed according to the institution where the study was conducted.

Author	Year	Institution	Description
León (34)	2021	Clinic	Develops the metrological assurance plan for the Intensive Care Unit and Surgical Ward of Clínica Universidad de La Sabana. The study shows flowcharts used for conformity assessment.
Duque (32)	2021	Clinic	Application of the Failure Modes and Effects Analysis (FMEA) methodology for medical equipment management in the Respiratory Therapy Service of a Healthcare Institution.
Rivera and Toloza (30)	2020	Clinic	Formulation of a proposal for a metrological assurance plan for IPS Oftalmosanitas SAS.
Gaviria and Romero (28)	2018	Clinic	Develop and implement a metrological assurance plan for two clinics of the Colsubsidio company.
Calambás (21)	2014	Clinical laboratory	Creates a guide for conducting metrological verification procedures in a clinical laboratory.
Montenegro (20)	2013	Clinic	Formulates a guide for evaluating medical devices within a clinic and compares existing methodologies for this purpose.
León-Ramentol et al. (29)	2017	University	Aim to demonstrate the importance of metrological assurance when implementing a quality management system at the Center for Immunology and Biological Products at the University of Medical Sciences of Camagüey.
Torres (33)	2021	Clinical laboratory	Develops an optimization plan for the metrological assurance program of the Public Health Department Laboratory of Antioquia, encompassing metrological monitoring, analysis based on national and international standards, design of an automation tool, implementation of improvements, and presentation of results.
García et al. (23)	2015	Clinic	Establish a model enabling the design and control of metrology processes in healthcare institutions by evaluating the calibration index, which determines the minimum calibration frequency required for various medical equipment. The model comprises three stages: analysis of a range of medical devices across eight healthcare entities, formulation of a metrological assurance plan, and establishment of necessary control mechanisms for these purposes.

### 3.4 Studies presenting implementation guidelines

Of the total, 31.3% (5/16) of the studies showed guidelines for implementing assurance systems (19, 22, 25–27). Four out of these five studies provided guides based on ISO 10012 and focused on metrological assurance (19, 22, 25, 27), while one presented a guide based on usability and electrical safety, focusing on users in small enterprises (26). Of these studies, three were conducted in Colombia (19, 25, 27), one in Costa Rica (22), and one in Brazil (26).

ISO 10012 is widely mentioned in the implementation guidelines for metrological assurance systems. From the analysis of Tables 2, 4, the relevance of ISO 17025 is also highlighted, implicitly incorporating certain aspects of ISO 9001. The latter standard proposes a process-based approach to address all organizational procedures to ensure quality.

**Table 4:** Studies on guidelines for the implementation of metrological assurance systems.

Country	Author	Type	Description
Colombia	Rodríguez (19)	Guideline	Formulate a methodology for the implementation of a metrological assurance plan for medical devices in healthcare institutions following the ISO 10012 framework.
Colombia	Rodríguez et al. (25)	Guideline	Develop a set of recommendations to follow during the implementation of a metrological assurance plan for medical devices in healthcare service providers, based on ISO 10012:2003.
Colombia	Ramírez Barrera et al. (27)	Guideline	Propose an integrated system for measurement assurance in medical equipment, based on ISO 10012:2003 and incorporating recommendations from PAHCE.
Costa Rica	Pacheco (22)	Guideline	Creates a guide facilitating the implementation of measurement assurance throughout the lifecycle of medical devices, in accordance with the ISO 10012:2003 standard and compliance with Costa Rican national legislation.
Brazil	Campese and Costa (26)	Guideline	Construct a guide for involving users in the verification process of medical devices, based on usability and electrical safety standards, tailored for small businesses.

### 3.5 Bias analysis

Two types of bias risk variables were not applicable to the selected documents, as they did not involve randomization or double-blind procedures, hence listed as “No information.” The majority (12/16) of the included studies had a low risk of bias, while three had insufficient information (Figure 1). The inter-observer agreement analysis was optimal (k=0.899) according to previous recommendations (35).

## 4 Discussion

This review of medical devices has showed the use of different standards, such as IEC 60601–1-6 for electromedical safety and IEC 62366 for usability. ISO 9000, ISO 9001, and ISO 15189 standards were mentioned for quality management by a third of the studies. Half of the studies were conducted in healthcare institutions, with only one study describing the use of eight national and international standards in Colombia, and discrepancies were identified in the use of national legislation and ISO 17025.

### 4.1 Strengths

This research stands out as the first systematic review comprehensively addressing metrological assurance systems for medical devices in Latin America. Unlike other studies that focus on specific aspects or evaluate institutions or countries (5–12), this analysis considers the various standards used in regional institutions. Understanding metrological assurance systems opens new opportunities to improve institutional design and functionality, promoting the creation of protocols or manuals that benefit the quality assurance of processes. Another strength of this research is its thorough information search, including grey literature, thereby expanding the database used for analysis.

### 4.2 Main findings

Based on the evidence found in Latin America, recommendations are made, such as using a reference standard to develop a metrological assurance system. In addition, fundamental characteristics for such a system are considered, including necessary input documentation, defining metrological needs, and outlining protocols (19, 22, 25, 27). These recommendations provide a solid foundation for the effective implementation of metrological assurance, ensuring the quality and accuracy of measurements in devices used in the healthcare field.

Across the reviewed studies, the implementation of metrological assurance systems typically occurred in institutions with a high number of equipment, such as clinics, laboratories, or universities. Due to this characteristic, there are few investigations that address this issue in smaller businesses, with Campese and Costa’s work (26) being an exception, aiming to involve users of small enterprises in the assurance process. However, it is noteworthy that ISO 9001 and ISO 10012 standards are easily adaptable to smaller organizations, including micro and small businesses. After analyzing the body of studies, fundamental components necessary for a metrological assurance system were identified, including proper identification of equipment used, recognition of their metrological needs (19, 25, 27), and the creation of a metrological assurance plan (19, 21, 25, 27). These components are tailored to meet the particularities of a small business, ensuring quality and precision in measurements within the healthcare field.

Our findings indicate that the most recommended standard for metrological assurance implementation is ISO 10012, and its citation frequency is comparable to that of ISO 17025 (36). ISO 17025 focuses on ensuring the technical competence of testing laboratories, assuring accuracy and reliability of test and calibration data. The standard encompasses key aspects of laboratory management, such as quality management system requirements, personnel competence, equipment calibration and maintenance, test methodology, and reporting results. However, this standard is designed for organizations with calibration laboratories and would only be applicable if a company can conduct internal calibrations on its devices (37). On the other hand, ISO 10012 (38) can be used to manage measurement processes and metrological confirmation of measurement equipment used to support and demonstrate compliance with metrological requirements in the context of medical device metrology. The standard specifies the quality management requirements of a measurement management system that an organization can employ as part of its overall management system, ensuring compliance with metrological requirements.

With the large number of small and microenterprises in the healthcare sector in Latin America, the implementation of standards and metrological assurance systems becomes even more relevant. This is essential to ensure measurement quality and, through that, the safety of diagnoses and patients, while also enhancing the competitiveness of these companies with procedures that can later be certified or accredited with ISO 9001 (16) or ISO 10012 (38).

Undoubtedly, the COVID-19 pandemic has emerged as an unparalleled event, delivering a seismic impact on the healthcare sector across various Latin American nations. In Peru, a country that has witnessed a surge in public health research over the past 50 years (39), there exists a palpable need for initiatives aimed at overseeing adherence to medical standards. This necessity arises as many research endeavors confront inherent limitations in their methodological approaches and outcome analyses (40). Simultaneously, the healthcare sector grapples with challenges such as a shortage of adequately trained personnel and constraints in the utilization of available technologies integral to daily clinical practice (41). Notably, certain technologies, including telemedicine, have demonstrated successful applications during the lockdown, holding the potential to enhance Clinical Risk Management, thereby elevating the Quality of Care and ensuring Patient Safety (42, 43). Nevertheless, it becomes imperative to diligently monitor the post-COVID landscape to propose regulatory frameworks that can effectively govern the seamless technological integration into comprehensive healthcare processes.

### 4.3 Limitations

Firstly, it is noted that the analysis was limited to four countries due to evidence availability. That did not allow us to perform a regional comparison with nations such as Chile, Argentina, Bolivia, Uruguay, or Paraguay, which lack available research on metrology. Secondly, the number of studies found (12/16) on metrology in Colombia might have influenced the overall results. While it is commendable that Colombia has a considerable number of biomedical engineering schools in nine universities (44), other countries like Peru have a more limited offering, with only two recent universities offering this program (45). This could impact the generalization of results, potentially predominantly reflecting the situation in Colombia. Therefore, it is essential to encourage research on metrology (46) across various Latin American countries to understand their realities and promote the development of metrological assurance systems for medical devices, thus ensuring quality in healthcare.

## 5 Conclusions and future directions

This study provides valuable insights into the implementation of metrology and quality control in the context of medical devices. The analysis of various applicable standards for medical devices, such as IEC 60601-1-6 and IEC 62366, along with ISO 9000, ISO 9001, ISO 17025, and ISO 10012, highlights the multifaceted approach necessary to ensure safety, accuracy, and reliability in healthcare technology. The research emphasizes the importance of incorporating both international and national legislation, and the inclusion of implementation guides based on ISO 10012, as well as the focus on small businesses, further contribute to the practicality and relevance of this research.

Addressing the challenges and requirements of metrology and quality assurance in medical devices, this study presents a valuable resource for advancing healthcare technology and improving patient care. Furthermore, it is crucial for both national and private institutions in each Latin American country to establish education and training programs focused on medical device metrology. This could ensure the quality of diagnoses in both larger hospitals and smaller organizations, benefiting patients. On the other hand, it is essential for academia and research centers to work on providing evidence and information availability, as well as conducting reference studies on medical device management and applicable metrological assurance plans. This will foster continuous advancement and improvement in the implementation of practices that ensure quality and accuracy in healthcare.

## Data availability statement

The original contributions presented in the study are included in the article/Supplementary material, further inquiries can be directed to the corresponding author.

## Author contributions

HF-V: Conceptualization, Data curation, Formal analysis, Investigation, Validation, Writing – original draft. DE-M: Conceptualization, Data curation, Investigation, Methodology, Supervision, Writing – original draft. MM-S: Formal analysis, Methodology, Software, Validation, Visualization, Writing – review & editing. HC-P: Data curation, Investigation, Project administration, Resources, Software, Writing – review & editing. JM-S: Conceptualization, Formal analysis, Methodology, Visualization, Writing – original draft, Writing – review & editing.
